# Supplementary material for: Study of Micro-Samples from the Open-Air Rock Art Site of Cueva de la Vieja (Alpera, Albacete, Spain) for Assessing the Performance of a Desalination Treatment
Source: Molecules. 2023 Aug 3;28(15):5854. doi: 10.3390/molecules28155854 (PMC10420967; doi:10.3390/molecules28155854)
Supplement: Supplementary file 1 [file molecules-28-05854-s001.zip › molecules-2508621-supplementary.pdf]

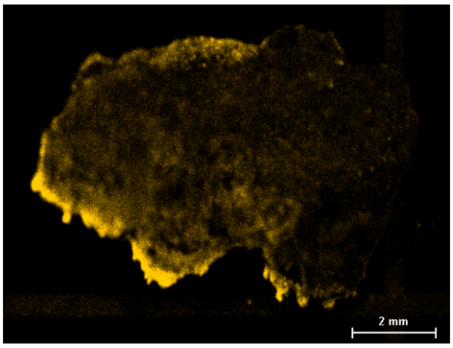

Figure S1. Distribution of sulfur in the interior face of the sample CuVi04.

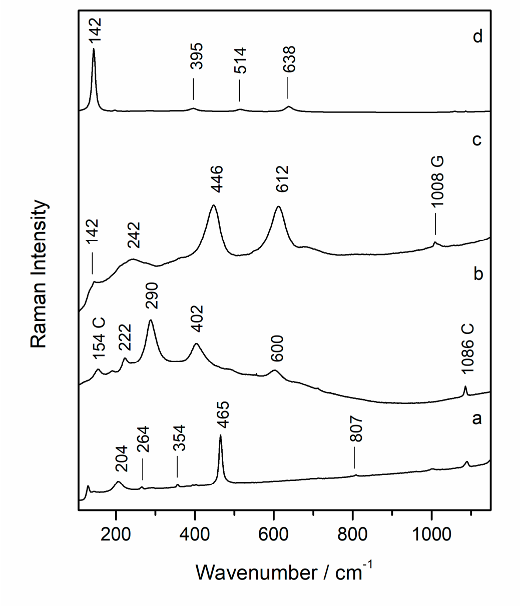

Figure S2. Raman spectra of quartz (a), hematite (b) with calcite, rutile with gypsum (c) and anatase (d).

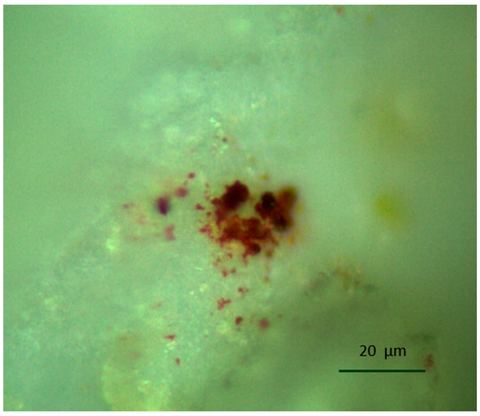

Figure S3. microscopic image of traces of hematite.

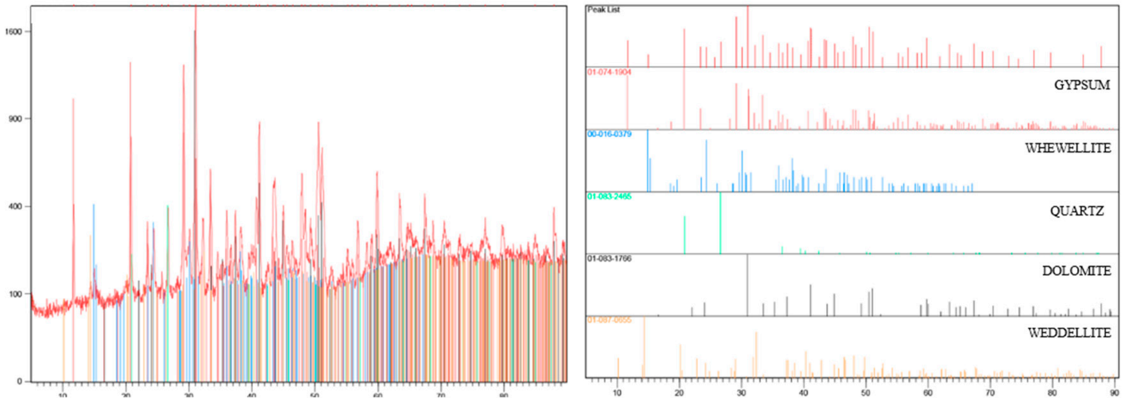

Figure S4. Diffractogram corresponding to the sample CuVi01 and assignment of compounds gupsum, whewellite, quartz, dolomite and weddellite.

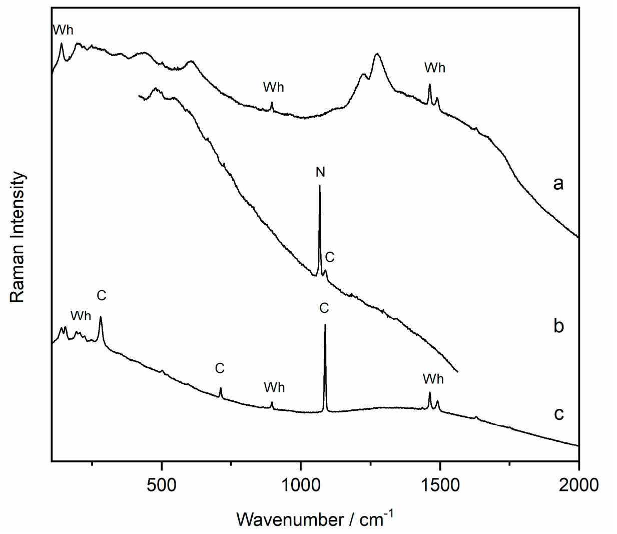

Figure S5. Raman spectra recorded in the samples taken from grey patinas show the presence of calcium oxalate whewellite and silicate compounds (a), calcium carbonate (C) with nitratine (N) (b) and with whewellite (c).

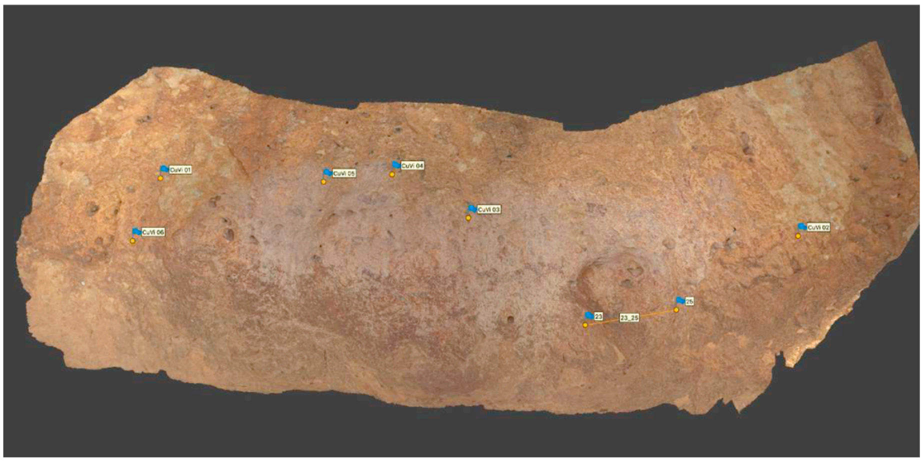

Figure S6. Painted panel from *Cueva de Vieja* where the sampling points are marked. The whitish layer is visible on the centre where the pictographs were made.
